# A quantitative survey of intern's knowledge of communication skills: an Iranian exploration

**DOI:** 10.1186/1472-6920-5-6

**Published:** 2005-02-08

**Authors:** Mohsen Tavakol, Sima Torabi, Owen D Lyne, Ali A Zeinaloo

**Affiliations:** 1 School of Education, Nottingham University, Wollaton Road, Nottingham, UK; 2 Ministry of Science, Research and Technology, Institute for research and planning in higher education, Iran; 3 Institute of Mathematics, Statistics and Actuarial Science, University of Kent, UK; 4 Educational Development Centre, Tehran University of Medical Science, Iran

## Abstract

**Background:**

It is a high priority that health care providers have effective communication skills. It has been well documented that the doctor-patient relationship is central to the delivery of high quality medical care, and it has been shown to affect patient satisfaction, to decrease the use of pain killers, to shorten hospital stays, to improve recovery from surgery and a variety of other biological, psychological and social outcomes. This study sought to quantify the current knowledge of interns in Iran about communication skills.

**Methods:**

A cross-sectional study using a self-report questionnaire was conducted among interns. Data analysis was based on 223 questionnaires. The internal consistency of the items was 0.8979.

**Results:**

Overall, knowledge levels were unsatisfactory. Results indicated that interns had a limited knowledge of communication skills, including identification of communication skills. In addition, there was a significant difference between the mean scores of interns on breaking bad news and sex education. The confidence of males about their communication skills was significantly higher than for females. Analysis of the total scores by age and sex showed that there was a statistically significant main effect for sex and the interaction with age was statistically significant. Free response comments of the interns are also discussed.

**Conclusions:**

It is argued that there is a real need for integrating a communication skills course, which is linked to the various different ethnic and religious backgrounds of interns, into Iranian medical curricula. Some recommendations are made and the limitations of the study are discussed.

## Background

The expectations of the public have been dramatically increased and the majority of them are familiar with their rights in the health care system. As a consequence, it is a high priority that health care providers have effective communication skills. It has been well documented that the doctor-patient relationship is central to the delivery of high quality medical care. It has been shown to affect patient satisfaction, to decrease the use of pain killers, to shorten hospital stays, to improve recovery from surgery and a variety of other biological, psychological and social outcomes [1-4]. Lack of knowledge of communication skills, or an inability to use them effectively, can be distressing and is potentially hazardous for patients. It may also be a cause of stress for medical students arriving on the ward for the first time [5]. There is a large body of evidence indicating the importance of students' knowledge of communication skills and [6,7] how behaviours learned from communication skills training transfer into the clinical setting and such training is known to have long term effects on students behaviour [8-11].

However, little is known about the importance of communication skills in the practice and training of doctors in Iran, where the culture differs greatly from that of the West. Sensitivity to religious matters is particularly important in Iranian doctor-patient relationships where Islam is more than a religion; it is a way of life. It controls politics, local laws, behaviour and many other aspects of daily life. It gives guidance in all spheres of human activity from birth to death. Therefore doctors coming into contact with religious patients need to be aware that there are numerous potential barriers to good communication [12].

A major criticism of current medical training in Iran is that communication skills have not been embedded in the curriculum of Iranian medical students, despite the richness and variety of evidence from elsewhere concerning the importance of communication skills. Concerns over poor doctor-patient communication amongst Iranian doctors led to an exploration of the current situation [13]. In this paper we investigate the knowledge level of interns about communication skills to gain a clearer picture of some challenges relating to health care promotion, especially patient satisfaction and adherence to treatment. Two questions guided the study: (a) How do interns assess their knowledge about communication skills? (b) Is there a significant difference between the level of knowledge among male and female interns?

## Methods

A quantitative survey was performed at Tehran University of Medical Science (TUMS). A cross-sectional study was conducted using a questionnaire administered to 235 interns. Anonymity was maintained throughout. The subjects received the self-administered questionnaire with a covering letter explaining the project and the subject's rights. 12 subjects did not return the questionnaire and an additional 7 subjects did not give their age and one person did not give his/her sex. Therefore data analysis was based on 223 questionnaires, but covariate-based analysis on fewer. The subjects were asked to complete the questionnaire without referring to source books.

The questionnaire consisted of three sections. The first section asked students to give personal details including the demographic items age and gender (summarised in Table 1). The second section is related to the educational items: subjects studied or attended in a specific course about communication skills (Table 2). The third section asked students to rate their knowledge of communication skills and, if they rated themselves higher than 5, discuss the item briefly in the space provided in order to assess their real knowledge with regard to that communication skill. In addition, they were encouraged to provide additional written comments on the questionnaire. The communication skills knowledge scale (CSKS) developed here consists of 10 items about communication skills. Each item is measured on a 10-point scale, ranging from 1 (low) to 10 (high).

**Table 1:** Distribution of background characteristics

Variables	Number	Percentages
Sex		
Male	132	59.5
Female	90	40.5
Total	222	100
Age		
Less than 25	114	52.8
25–30	96	44.4
More than 30	6	2.8
Total	216	100

**Table 2:** Percentage response to educational items by interns

Educational item	Yes	No
	%	%
1. Have you studied a paper in relation to communication skills? (n = 219)	21.9	78.1
2. Have you formally attended communication skills courses? (n = 221)	8.6	91.4

The choice of items was based on the communication skills an intern will need. All items were verified and subjected to content validation by three major experts in communication skills. These experts were given copies of the CSKS and the purpose and objectives of the study. They then evaluated the CSKS on an individual basis. Comparisons were made between these evaluations and the authors then made some minor changes within the CSKS. The CSKS had a high internal consistency (*Cronbach alpha *= 0.8979).

The validity of the CSKS can only be examined through logical rather than empirical means. Since the CSKS was not compared to a standardised test, it was impossible to obtain a numerical estimate of the validity of the test. However, based on logical means, i.e., a respectable *Cronbach alpha *and high inter-rater agreements on each item, the authors believe that the test is valid. The questions and responses have been translated from Persian into English for this paper.

## Results

The potential score range from the 10-item CSKS (by summing all 10 item scores) is 10 to 100, with 10 indicating low knowledge. Analysis of the total scores produced a mean score of 51.30 [95 per cent confidence interval (CI) 49.05–53.55]. The subjects' performance on the CSKS suggests a knowledge deficit in communication skills. The mean scores for males and females were respectively 53.6 and 48.2 (P = 0.02). The vast majority of interns (78.1%) had not studied a paper on communication skills. When asked whether they had formally attended communication skills courses, 91.4% of interns reported "no". Of the few interns who reported "yes", these interns specified courses such as CPR, injections and semiology (Table 3), which are not formal communication skills courses.

**Table 3:** Courses of communication skills training reported by interns

Courses	Number
CPR	2
EBM	1
Ethics	4
Health	2
Injection	1
Skills lab	3
Semiology	3
Workshop	1
Total	17

The analysis of the scores by topic is shown in table 4. The possible range of scores for each item was 1 to 10. Mean scores for topics ranged from 2.8 to 6.1. Interns were most confident on "giving and receiving information", and the least confident on "sex education".

**Table 4:** Analysis of results by communication skill

Topics	Mean		P Value
	Male (SD)	Female (SD)	
Breaking bad news	4.6 (2.0)	3.7 (2.0)	0.02
Dealing with anger/difficult patient	5.0 (2.4)	4.4 (2.2)	0.81
Demonstration of empathy	5.3 (2.3)	5.5 (2.4)	0.56
Giving and receiving information	6.1 (2.3)	5.9 (2.2)	0.46
Non-verbal communication skills	5.3 (2.5)	5.0 (2.0)	0.37
Dealing with patient perception	5.8 (2.4)	5.4 (2.1)	0.22
Shared decision making	5.7 (2.2)	5.4 (2.3)	0.28
Patient-oriented interviewing	5.4 (2.3)	5.3 (2.3)	0.17
Sex education	4.6 (2.7)	2.8 (1.9)	0.00
Closing skills	5.4 (2.4)	4.8 (2.4)	0.08
Total	53.6 (17.4)	48.2 (15.8)	0.02

A two-way between-groups analysis was conducted to explore the impact of sex and age on levels of knowledge, as measured by the CSKS. Subjects were divided into two groups according to their age (less than 25 years, or 25 years and above). There was a statistically significant main effect for sex [F (1, 212) = 4.90, p = 0.02] and the interaction effect [F (1, 212) = 4.06, p = 0.04) did reach statistical significance. However the effect size was small (eta squared = 0.02). The young male interns were more confident than average, while the young female interns were less confident.

Free responses included the following comments:

'*Nobody has trained us about communication skills. Our knowledge in respect of communication skills is very poor. Your items show that we are very far behind other countries. Our universities are not as advanced as other universities'*.

*'I feel we are not familiar with the ABC of communication skills'*.

*'A good guide to communication skills needed'*.

*'I feel communication skills would be an excellent course since it gives us an idea of how we can handle bad news'*.

*'Attending doctors are not totally familiar with the aims and use of communication skills in the clinical setting'*.

'All our courses only focus on biological issues rather than psychosocial issues'

## Limitations

There were a number of limitations to this study.

1. The CSKS has not been normed for a population of interns.

2. Criterion-related validity of the CSKS was not determined, although content validity was established on the instrument.

3. Since it is a self-assessed questionnaire, these may be problems with bias, such as prestige bias.

## Discussion

The very high response rate (95%) of this questionnaire may have reflected general interest, or may have resulted from the advantages of self-assessment which itself may improve performance. The results on the CSKS show that basic knowledge of interns in Iran about communication skills is limited. Researchers have reported similar findings in other countries which reveal a deficit in the knowledge of doctors about communication skills [14]. The importance of communication skills has long been acknowledged in general practice training [15] and the need to teach communication skills formally, as part of British undergraduate medical education, has also been recognised [16]. In Iran, interns' knowledge deficiency may be attributed to the fact that interns have never been trained to consult in the general practice setting, and their skills are limited to making value judgements, often using the only available criterion, comparison with their own style [13]. This approach to a patient is not cost effective and may lead to negative health outcomes such as patient dissatisfaction, poor adherence to treatment and medical errors [17]. A few students reported their attendance at courses such as EBM, semiology, skills lab or CPR, which have no relation with communication skills training. This indicates that students are not familiar with the tasks of communication skills [18].

The vast majority of research studies have been conducted on the outcome of communication skills in the practice and training of doctors in western countries. Even here, despite doctors trained in communication skills and the advocacy of the use of a patient-oriented approach, some evidence suggests that there are difficulties in practice [19,20]. However, research has demonstrated that communication skills training intervention using behavioural, cognitive and affective domains can increase not only potentially beneficial and effective interviewing styles, but also alter attitudes and confer other benefits [9,21].

The results of the study show that there were significant differences between males and females with regard to their reported knowledge of the main communication skills. Women were less confident of their skills. The deficit may partly be an artefact of an inadvertent prestige bias of the male students. The deficit is particularly notable in sex education. There are three possible explanations for this. Firstly, in general, Iranian female interns are very shy to ask patients about sexual issues. Therefore they may feel that sex education skills have no implications for their practice and hence pay less attention to sex education training. Secondly, it may be a systematic error in female respondents, i.e. they may be shy to discuss their knowledge about sex education skills rather than lack knowledge. Thirdly, in the past, sex education was regarded as a taboo in Iran and was not available in schools, especially for girls [22]. This perhaps acts as an inhibitory factor on the basic knowledge of sex education. Within this context, there is no evidence that shows similar results for gender difference on the knowledge of sex education in the practice and training of doctors.

The results on the CSKS suggest that there are areas of weakness in the communication skills confidence of interns, particularly in breaking bad news. While it is well recognised that delivering bad news is a difficult task that requires skills and sensitivity [23], both female interns and male interns reported that their confidence in breaking bad news is low, especially the female interns. While the interns commented on the need to improve medical students' communication skills, it seems that guidelines on delivering bad news to patients and patients' family members have not been seriously taken into consideration in the practice and training of doctors in Iran. This could be due to interns possessing deep fears regarding delivering bad news to patients' family members, or because they are unaware of the general guidelines about delivering bad news [24]. Three studies which have attempted to address residents' perception of delivering bad news indicate that residents had experienced discomfort with psychosocial issues related to the conveyance of bad news, such as personal fears and different perceptions of bad news [25-27].

There is a significant difference between the mean score of the interns on breaking bad news. The female interns have reported lower confidence than the male interns. The deficit could be an inadvertent prestige bias of the male students. However, to our knowledge, there is no evidence that underpin such finding. Although Orlander et al's work [28] demonstrated there were no significant differences between males and females with regard to the type of bad news, residents' knowledge with regard to breaking bad news was not reported by the authors. Therefore, some empirical research is essential.

Given the poor levels of confidence about communication skills, particularly sex education skills, revealed in this study, it is concluded that educational programmes are necessary. In sex education skills training, given the complex interplay of cultural and religious beliefs in Iran, particular attention must be paid to multicultural and religious issues. Therefore, further work is needed on gender education and stereotypes in sex education; learning styles; the 'hidden curriculum'; and how far medical schools make organisational and administrative arrangements on the basis of gender and the implication for female and male interns.

The enthusiastic response to the questionnaires may suggest that medicine is accepting the need for developing communication skills within the medical curriculum. Medical education in Iran must respond to this challenge.

Finally, our findings may be somewhat limited in generalisability because they are derived from only one medical school in Iran. Self-assessment data may suffer from biases such as prestige bias. Despite these caveats, the authors believe the data to be an accurate reflection of current practice in Iran, based on the Iranian authors training experiences, and consistency with previous accounts.

## Conclusions

Whilst the approach to this research has been shaped by a government-recognised health need, the authors recognise the need for, and welcome, further examination of these findings from multiple perspectives, especially with regards to ethnicity and social issues. Since not enough attention has been focused on individuals as makers of health as a service rather than customers of health care services, it is strongly recommended, therefore, that medical students be trained in the context of psychosocial issues that may influence health behaviour, as has been indicated by one of the participants. It is particularly important that this type of approach be incorporated into the curricula of medical training. This may assist in transferring from the disease-oriented to the patient-oriented approach and ultimately lead to patients understanding more and taking greater responsibility for their own health.

## Competing interests

The author(s) declare that they have no competing interests.

## Authors' contributions

MT and ST carried out the conception, design, initial analysis and interpretation of the data. MT drafted the paper. ODL was involved in revising the draft critically, revising the statistical analysis and gave final approval of the version to be published. AAZ contributed to the collection of data and the reviewing of the manuscript.

## Pre-publication history

The pre-publication history for this paper can be accessed here:


